# The CoREST Complex Regulates Alternative Splicing by the Transcriptional Regulation of RNA Processing Genes in Melanoma Cells

**DOI:** 10.3390/cells14211699

**Published:** 2025-10-29

**Authors:** Abdul Aziz Khan, Ariel A. Aptekmann, Dongkook Min, Michael C. Povelaitis, Sunmi Lee, Byungwoo Ryu

**Affiliations:** 1 Center for Discovery and Innovation, Hackensack Meridian Health, Nutley, NJ 07110, USA; 2 Department of Medical Science, Hackensack Meridian School of Medicine, Nutley, NJ 07110, USA; 3 Lombardi Comprehensive Cancer Center, Georgetown University, Washington, DC 20057, USA

**Keywords:** CoREST complex, NOLC1/NOPP140, c-MYC, RNA splicing, melanoma

## Abstract

RNA maturation, particularly splicing, depends on coordinated actions of RNA-binding proteins through post-transcriptional processing and constitutes a central mechanism of gene regulation. Aberrant splicing is associated with various diseases, including cancer. Here, we show that the CoREST complex, in coordination with c-MYC, transcriptionally regulates a subset of RNA processing genes, including those encoding essential small nuclear ribonucleoproteins (snRNPs) required for proper spliceosome function. Genetic depletion or the pharmacological inhibition of the CoREST complex in melanoma cells disrupted spliceosome activity, leading to widespread changes in alternative mRNA isoform expression and reduced cell viability. These splicing alterations were associated with changes in the 2′-O-methylation (Nm) of U1 snRNA, a modification critical for spliceosomal function. The ectopic expression of the nucleolar protein NOLC1, a downstream target of the CoREST complex and known for its role in ribosomal RNA processing, partially rescued viability, splicing patterns, and U1 snRNA methylation in CoREST-deficient melanoma cells. Conversely, NOLC1 depletion sensitized melanoma cells to the MEK inhibitor trametinib, a clinical drug approved for treating advanced melanoma. Together, these findings uncover a novel CoREST-NOLC1 axis which is a transcriptional regulatory mechanism playing a significant role in RNA splicing, highlighting that NOLC1 is a downstream effector of the CoREST complex and a potential therapeutic target for melanoma treatment.

## 1. Introduction

The CoREST complex is a chromatin-modifying multi-subunit transcription regulator consisting of two histone-modifying enzymes, class I histone deacetylase 1 or 2 (HDAC1/2) and lysine-specific histone demethylase 1A (LSD1/KDM1A), and CoREST1/RCOR1 that bridges the two enzymes [1,2,3,4]. The CoREST complex transcriptionally represses genes by erasing active histone marks, including histone acetylation, and mono- and di-methylations at histone H3 lysine 4 (H3K4me1/2) [2,3,4,5]. CoREST1 is one of the first proteins characterized to silence neuronal genes with the help of REST/NRSF (RE1 Silencing Transcription factor/Neural-Restrictive Silencer Factor), a DNA-binding transcription factor that recruits CoREST1 to repress neuronal-specific gene transcription in non-neuronal cells for developmental processes [1,5]. The CoREST complex preferentially binds to proximal promoters of highly expressed genes at transcriptionally permissive chromatin loci in euchromatin and acts as a transcription rheostat [6]. The CoREST complex also interacts with transcription co-factors, such as hormone (androgen and estrogen) receptors and the NeuroD1 transcription factor, and is able to promote their target gene transcription [7,8,9], highlighting its broader role as a transcriptional modulator in mammalian cells.

Post-transcriptional RNA processing in mammalian cells comprises a coordinated series of biochemical modifications to precursor messenger RNA (pre-mRNA), including 5′-end capping, 3′-end polyadenylation, and splicing [10]. Splicing precisely removes non-coding introns and joins coding exons to generate mature mRNA isoforms, enabling proteomic diversity through alternative splicing [11,12]. The dysregulation of splicing machinery or aberrant alternative splicing can disrupt normal cellular functions and contribute to oncogenesis by promoting the expression of oncogenic isoforms, evading apoptosis, and enhancing proliferation, thereby playing a crucial role in cancer development and progression [13,14]. Studies have shown an elevated expression of components of the CoREST complex, such as RCOR1 and LSD1, in various tumor cells compared with normal cells [15,16,17]. This overexpression is often associated with a poor prognosis and contributes to tumor development and progression.

In this study, we observed that post-transcriptional RNA processing genes are downregulated with the depletion of RCOR1 in melanoma cell lines, with significant changes in the alternative splicing patterns. Many of these genes are transcriptionally regulated in coordination with the oncogenic transcription factor MYC. In particular, we found that one of the CoREST complex and MYC target genes encoding nucleolar and coiled-body phosphoprotein 1 (NOLC1, also called NOPP140) plays a significant role in the regulation of mRNA splicing via the 2′-O-methylation (Nm) of U1 snRNA, a critical component of the U1 spliceosome. This suggests a potential mechanistic basis for how the CoREST complex controls cancer-specific mRNA splicing in melanoma cells.

## 2. Materials and Methods

### 2.1. Cell Culture

The cell lines used in this study were cultured in DMEM supplemented with 10% FBS and 1% penicillin/streptomycin at 37 °C with 5% CO2 in a humidified incubator. WM983B and 1205Lu cells were obtained from M. Herlyn (The Wistar Institute, Philadelphia, PA USA). All other cells were purchased from ATCC (Manassas, VA, USA) and tested to be free of mycoplasma.

### 2.2. Plasmid Construction and Lentivirus Production

The knockdowns were performed using lentiviral plasmid pLKO.1-TRC (Addgene_10878, Watertown, MA, USA). The plasmid was digested with EcoRI (NEB, Ipswich, MA, USA) and AgeI (NEB, Ipswich, MA, USA). The annealed shRNA oligos for the target genes were cloned into the EcoRI and AgeI sites of pLKO.1 vector and ligated with Ligase mixture (TAKARA, San Jose, CA, USA). The shRNA clones were confirmed by Sanger’s sequencing (Azenta, South Plainfield, NJ, USA). For the NOLC1 promoter activity confirmation, a ~1 kb regulatory region was cloned into NheI/HindIII sites of the pGL4.10 vector (Promega, Fitchburg, WI, USA). Primer sequences for this cloning are shown in Table S1. The clones were confirmed by Sanger’s sequencing (Azenta). Transient transfections were conducted in melanoma cell lines WM983B and A375 using Lipofectamine transfection reagent (Invitrogen, Waltham, MA, USA). Cells were co-transfected with 2 µg of firefly pGL4.10 reporter and 200 ng of *Renilla* luciferase reporter plasmid (Promega). After 24 h the cells were treated with 1 µM corin and incubated for 24 h. The cells were harvested, and the luciferase activity was measured using the Dual-Luciferase^TM^ reporter assay system (Promega). Firefly luciferase values were divided by *Renilla* luciferase values and the data from three reactions were averaged.

For the ectopic expression of proteins, the genes were PCR amplified from cDNA, digested with BamHI (NEB) and NotI (NEB), and cloned into a pCDH-EF1α-puro vector under the EF1α promoter and puromycin selection marker with an HA or FLAG tag. The clones were confirmed by Sanger’s sequencing (Azenta). The RCOR1 clone was purchased from a commercial company (GeneCopoeia_U0745, Rockville, MD, USA). The mutant MYC clones were amplified with primers containing the mutant amino acid sequences from the WT MYC clones and confirmed by Sanger’s sequencing (Azenta). For the construction of lentiviral particles, Lenti-X-293T cells were transfected with ORF or shRNA vectors with packaging plasmids Pax2, PMD2.G at 3:2:1 using Lipofectamine 2000 (Invitrogen). The viral particles were harvested 48 and 72 h post transfection and filtered through a 0.45 µm filter. Cells were transduced with viral particles and selected with 1 µM puromycin for at least 72 h.

### 2.3. RNA Sequencing and Analysis

RNA-seq data were re-analyzed from our previously published article [18]. Briefly, the sequence reads were trimmed to remove possible adapter sequences and nucleotides of poor quality using Trimmomatic v.0.39. The trimmed reads were mapped to the Homo sapiens GRCh39 reference genome available on ENSEMBL using STAR aligner v.2.7.1b. Gene hit counts and unique gene hit counts were calculated using FeatureCounts from the Subread package v.2.0.3. Gene counts were further analyzed to test for differential expression using R package DESeq2 [19] contrasting with RCOR1 KD or Corin treatment. For GSEA, the differentially expressed genes (DEGs) between RCOR1 knockdown and the control in each cell line were chosen up to 10 reads, and log2 fold changes between the samples were ranked from top to bottom. The data set was analyzed using the software program GSEA4.3.2 downloaded from the Broad Institute, and the enriched gene sets were identified based on the human Molecular Signature Database (MSigDB v2023.1.Hs). Differential exon usage analysis was performed using R package DEXseq (version 4.5.0) [20] by following standard protocol.

### 2.4. ChIP Sequencing

A ChIP assay was performed as described previously [21]. Briefly, cells were crosslinked with 1% formaldehyde for 10 min, and the reaction was stopped with a final concentration of 0.137M Glycine for 5 min followed by two washes with ice-cold PBS. Cell nuclei were extracted with buffer 1 (10 mM HEPES [pH 6.5], 0.25% Triton X-100, 10 mM EDTA, 0.5 mM EGTA, and 1 mM PMSF) and buffer 2 (10 mM HEPES [pH 6.5], 200 mM NaCl, 1 mM EDTA, 0.5 mM EGTA, and 1 mM PMSF). The nuclei pellet was resuspended in RIPA buffer and sonicated to a range of 300~500 bp with an M220 focused-ultrasonicator (Covaris). The sonicated chromatin was incubated with 5 µg of indicated antibodies followed by A/G magnetic beads (Fisher Scientific, Waltham, MA, USA) and incubated overnight at 4 °C with overhead rotation. The complex was washed once with buffer 1 (0.1% SDS, 1% T-X-100, 2 mM EDTA, 150 mM NaCl, and 20 mM Tris-HCl (pH 8.1)) for 10 min, twice with buffer 2 (0.1% SDS, 1X T-X-100, 2 mM EDTA, 500 mM NaCl, and 20 mM Tris-HCl (pH 8.1)), twice with buffer 3 (0.25 M LiCl, 1%NP-40, 1% Sodium deoxycholate, 1 mM EDTA, and 10 mM Tris-HCl), and finally twice with 1X TE buffer. A total of 10% of the sonicated chromatin was used as input. ChIP samples were reverse crosslinked with proteinase K for 12 h at 65 °C. The DNA was purified using the phenol/chloroform method and sequenced at GENEWIZ (South Plainfield, NJ, USA). We performed a preliminary quality check of the ChIP-seq data sets using FASTQC, a quality control tool for high-throughput sequence data, then aligned the reads to the Homo sapiens genome and annotations version GRCh39 available on ENSEMBL using the standard protocol and parameters from STAR aligner v2.7.1b. We called peaks from the aligned read files for both RCOR1 and MYC datasets using MACS version 3 and “callpeaks” function [22], and kept all peaks using the IgG data set as control with all standard parameters except for “-q 0.01” for the downstream analysis. We further identified sites with peaks in common between RCOR1 and MYC using INTERVENE [23].

Other bioinformatics analysis: Gene ontology (GO) term analysis with RCOR1 and MYC peak sequences were performed using PANTHER [24] over-representation analysis and GO database released 10 May 2023.

### 2.5. Re-ChIP Analysis

The chromatin for ChIP-re-ChIP was prepared as above for ChIP-Seq. After performing ChIP, the immunoprecipitated chromatin was eluted with an elution buffer (1 mM EDTA, 1%SDS, and 10 mM DTT) and incubated at 65 °C for 10 min. The eluted product was diluted ten times in the RIPA buffer excluding SDS and divided into two parts. To one part, the antibody–beads complex was added, and to another half, only beads were added to use as a control and incubated overnight with overhead rotation at 4 °C. The chromatin was washed again with buffer 1, buffer 2, buffer 3, and TE buffer as mentioned above. The DNA was isolated by incubating the complex with proteinase K for 12 h at 65 °C and purified with phenol/chloroform and ethanol precipitation.

### 2.6. Cell Growth Assay

Cell growth assay was performed using CellTiter-Glo 2.0 reagent (Promega, Fitchburg, WI, USA) according to the manufacturer’s instructions. Briefly, cells were grown in 96-well plates and a volume of CellTiter-Glo 2.0 reagent was added. The content was mixed on an orbital shaker for 2 min to lyse the cells and then incubated for 10 min at room temperature to stabilize the luminescent signal.

### 2.7. Immunoblot and Antibodies

Cells were lysed in RIPA buffer (10 mM Tris, 1 mM EDTA, 0.5 mM EGTA, 1% T-X-100, 0.1% sodium deoxycholate, 0.1% SDS, and 140 mM NaCl) followed by incubation at 4 °C with overhead rotation. The cell debris was removed by centrifugation. The protein was quantified by BCA assay (Fisher Scientific, Waltham, MA, USA). Protein was resolved on SDS-PAGE and immunoblotting was carried out with indicated antibodies. The primary antibodies used in this study are listed in Table S1.

### 2.8. RT-PCR

RNA was isolated by TRIzol reagent (Life Technologies) according to the manufacturer’s instructions followed by DNase I (Invitrogen) treatment. RNA was purified by phenol/chloroform and dissolved in nuclease-free water. RNA was reverse transcribed using the SuperScript III first-strand synthesis system (ThermoFisher Scientific) or cDNA synthesis kit (Applied Biosystems, Foster City, CA, USA). The cDNA was subjected to semiquantitative RT-PCR using the SYBR green master mix (TOYOBO, New York, NY, USA). For the exon usage confirmation, the primers were designed based on RNA-seq exon usage regions and amplified by the KOD PCR master mix (TOYOBO). The PCR products were run on agarose gel. The list of primers used in the PCR is indicated in Table S1.

### 2.9. RTL-P

For the detection of 2-O’՜-methylation, the RTL-P (Reverse Transcription at Low dNTP concentrations followed by PCR) was performed as described previously [25] with slight modifications. Briefly, cDNA was made with 4 µM dNTPs concentration and gene-specific primer by using 200 U of M-MLV reverse transcriptase (Promega). The reaction was incubated at 42 °C for 1 h and enzyme deactivated at 85 °C for 5 min. The PCR was performed with the conventional method by using the low dNTPs template.

### 2.10. Public Resources and Data Mining

The comparison of the differential gene expression of NOLC1 based on tumor and normal skin from the Cancer Genome Atlas (TCGA; https://www.cancer.gov/tcga (accessed on 3 May 2025)) and The Genome-Tissue Expression (GTEx) project (https://gtexportal.org/home/ (accessed on 27 March 2025)) [26] databases was generated by GEPIA2 (http://gepia2.cancer-pku.cn/#index (accessed on 27 March 2025)) [27]. RNA-seq data of the RCOR1 and NOLC1 and clinical information including overall survival rates, vital status, and expression levels based on TPMs were downloaded from the Genomic Data Commons (GDC) data portal (https://portal.gdc.cancer.gov/ (accessed on 15 April2025)).

### 2.11. Statistical Analysis

Statistical analysis was performed with GraphPad Prism version 8 (GraphPad Software Inc., San Diego, CA, USA) or an MS Office Excel spreadsheet. Data are presented as the geomean ± SD of replicated experiments. Significance was determined using an paired/unpaired Student’s *t* test, ANOVA test, or log-rank test (Kaplan–Meier’s curves). The correlation coefficient was calculated using Spearman’s or Pearson’s methods.

## 3. Results

### 3.1. The CoREST Complex Regulates Genes Mediating Post-Transcriptional RNA Processing in Melanoma

**Figure 1:**
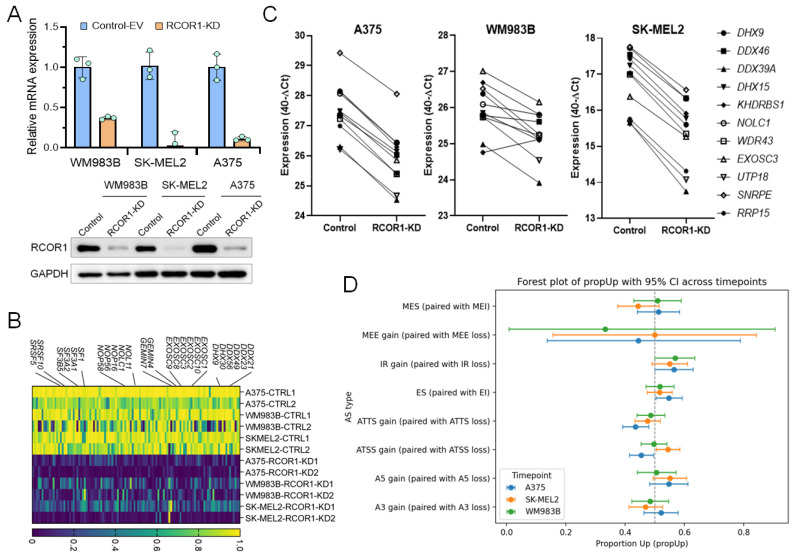
CoREST complex transcriptionally upregulates genes functionally associated with RNA processing in melanoma. (**A**) RT-PCR (upper panel) and Western blot (lower panel) showing the depletion of RCOR1 in three melanoma cell lines. (**B**) Expression heatmap of genes differentially expressed in RCOR1 KD. Genes were selected from the RNA processing gene set (GO:0006367) that are commonly downregulated in all three melanoma cell lines (WM983B, A375, and SK-MEL2, *n* = 3) with RCOR1 KD. The legend indicates normalized fractions of TPM values (control-EV vs. RCOR1-KD) with a scale of 0 (minimum) to 1 (maximum). (**C**) Semi-quantitative RT-PCR assay of selected genes from the RNA processing gene set (GO:00006396) that are downregulated in all three melanoma cell lines (A375, SK-MEL2, and WM983B) with RCOR1-KD compared with isogenic control-EV cell lines. (**D**) Splicing analysis of the significantly altered genes using IsoformSwitchAnalyzer on the RNA-seq by alternative splicing in RCOR1-KD cells compared with control-KD cells. A3 (alternative 3′ splice site), A5 (alternative 5′ splice site), ATSS (alternative transcription start site), ATTS (alternative transcription termination site), ES (exon skipping), IR (intron retention), MEE (mutually exclusive exons), and MES (mutually exclusive splicing/exons).

To gain insights into the functional roles of the CoREST complex in cancer cells, we knocked down RCOR1 by shRNA in three melanoma cell lines (A375, WM983B, and SK-MEL2) and confirmed the downregulation by RT-PCR and Western blot (Figure 1A) followed by transcriptomic analysis by RNA-sequencing. Differentially expressed genes (DEGs) were identified by comparison with control-EV (empty vector) cells. Consistent with its established function as a transcriptional repressor, decreased RCOR1 expression primarily led to the upregulation of numerous genes. However, a significant number of genes were also downregulated in all three cell lines, reflecting the recently recognized dual regulatory role of the CoREST complex [28,29].

To identify common gene signatures regulated by the CoREST complex, we performed a Gene Set Enrichment Analysis (GSEA) paired with the human Molecular Signature Database (MSigDB v2023.1.Hs). While upregulated genes were enriched for immune- and inflammation-related gene sets (Supplementary Figure S1A,B), the most highly enriched gene sets among the downregulated genes fell into two distinct functional groups. One group was associated with chromosome regulation, including DNA replication, chromosome segregation, and sister chromatid segregation. The other group was associated with post-transcriptional RNA processing, specifically ribonucleoprotein complex biogenesis and RNA splicing via transesterification (manuscript in review).

Among the genes notably downregulated across all three RCOR1-KD melanoma cell lines, we noted several core components of key RNA processing complexes. These included several core components of spliceosome complexes (*SF1*, *SF3A1/2/3*, *SF3B5*, *SRSF5/10*, and *GEMIN4/7*), DEAD/H-box RNA helicases (*DDX21*, *DDX23*, *DDX49*, *DHX9*, and *DHX30*), nuclear RNA exosome complexes (*EXOSC1*, *EXOSC2*, *EXOSC3*, *EXOSC8*, *EXOSC9*, and *EXOSC10*), and small nucleolar ribonucleoproteins (snoRNPs) which are involved in both spliceosome and ribosome assembly and biogenesis (*NOL11*, *NOLC1*, *NOP56*, and *NOP58*) (Figure 1B). We confirmed the downregulation of these genes by semi-qRT-PCR (Figure 1C), suggesting dysregulation in a broad range of post-transcriptional RNA processing genes by the CoREST complex in melanoma cells. We performed an alternative splicing analysis using IsoformSwitchAnalyzer on the RNA-seq of the three cell lines of this analysis and we found many genes significantly altered by alternative splicing (A375: 921, SK-Mel2: 998, Mel9: 824). Although a large number of genes were affected, the gene overlap between the cell lines was as low as 10%. This high altered gene count suggests a general mechanism influencing alternative splicing rather than a targeted, cell-line-specific effect. We then compared the type of effect and noticed for the three cell lines a consistent change in intron retention (Figure 1D). To further validate these findings, we re-analyzed our previous RNA-seq data from cells treated with the CoREST complex inhibitor, Corin [18]. Corin treatment caused a strong downregulation of RNA processing genes (Figure S2A) and changes in mRNA isoforms, recapitulating the effect of genetic RCOR1 KD (Figure S2B). These results collectively suggest that the CoREST complex transcriptionally regulates RNA processing genes and plays a crucial role in alternative splicing.

### 3.2. CoREST and MYC Transcriptionally Regulate the RNA Processing Genes

The role of the CoREST complex is well established as a transcriptional repressor [30,31], but its gene activation function has also been recognized [29]. For transcriptional regulation, the CoREST complex partners with other proteins to regulate transcription. For example, RCOR1, a core component of the complex, interacts with the Hsp70 protein to repress both the HSF1- and heat shock-dependent transcriptional activation of the *Hsp70* promoter [32]. We have recently identified a novel mechanism in which the CoREST complex interacts with and stabilizes the oncoprotein MYC through lysine deacetylation (manuscript in review).

**Figure 2:**
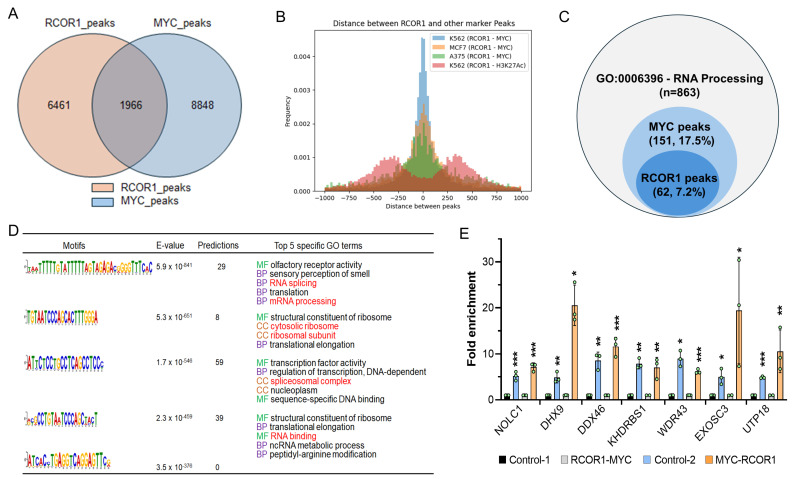
CoREST complex cooperates with MYC to control the transcription of a subset of genes involved in the biological steps of RNA processing in melanoma. (**A**) Venn diagram showing the counts of genes occupied by RCOR1 and MYC within ±2.0 kbps from the TSS of genes in the A375 cell genome. (**B**) RCOR1 and MYC ChIP-seq peak distance related to the TSS in the A375, K562, and MCF7 cells genomes. (**C**) Venn diagram showing the counts of genes harboring RCOR1 ChIP-seq peaks and MYC ChIP-seq peaks at the regulatory regions of RNA processing genes (GO:00006396). (**D**) RCOR1-binding motifs and predicted GO terms that are specific to the motif-binding factor RCOR1 in A375 melanoma cells. (**E**) Re-ChIP PCR analysis at the proximal promoter regions of genes selected from the 62 genes co-regulated by the CoREST complex and MYC. The CoREST-MYC fold change has been calculated based on control 1 (CoREST-IgG) and the MYC-CoREST fold enrichment was calculated based on control 2 (MYC-IgG). The data indicates fold-change enrichment. Error bars represent geomean ± SD of three biological replicates and a two-sided Student’s *t*-test was used. * *p* < 0.05, ** *p* < 0.01, and *** *p* < 0.001.

To explore the possibility of the CoREST complex and MYC cooperatively playing a regulatory role in the transcriptional activation of RNA processing genes, we performed RCOR1 and MYC chromatin immunoprecipitation sequencing (ChIP-seq) for both RCOR1 and MYC in A375 melanoma cells. We found that 30% of the RCOR1 ChIP-seq peaks (1966 out of 6461 peaks) within gene transcription regulatory regions, defined as ±2.0 kb from the transcription start site (TSS), were also co-localized with MYC ChIP-seq peaks (Figure 2A). The intervals between the two sequence motifs were searched. Most RCOR1- and MYC-binding motifs were located within a span of a nucleosome length of DNA (approximately 180 bp), which was also true in two different types of cancer cell lines: lymphoblast (K562) and breast cancer (MCF7) (Figure 2B). To further corroborate the role of the CoREST complex in the transcriptional regulation of RNA processing genes, both RCOR1- and MYC-binding peaks in the proximal regulatory regions of genes known for regulating RNA processing (GO:0006396) were determined. A subset of genes (62 out of 863 genes, 7.2%) harbored RCOR1-binding motifs, while 151 genes (17.5%) harbored MYC-binding peaks (Figure 2C).

To explore the possibility of RCOR1 regulating RNA processing, we performed a gene ontology (GO) term analysis using the GOMo (Gene Ontology for Motifs) program (MEME suite version 5.0.5) [33] to identify biological processes potentially regulated by the genes controlled by the RCOR1 motifs. We found that most gene sets regulated by the RCOR1-binding motifs are involved in the biological role of RNA processing, including RNA splicing and ribosome biogenesis (Figure 2D). Interestingly, all the RCOR1-bound gene promoter regions are also co-occupied by MYC (Figure 2C). A few genes selected from the list of 62 genes harboring the RCOR1 and MYC peaks by ChIP sequencing analysis are shown in Figure S3A. The binding of the CoREST complex and MYC was confirmed by ChIP PCR (Figure S3B). Similarly, the co-binding of CoREST and MYC was corroborated by a Re-ChIP assay (Figure 2E) at the proximal regulatory regions of the selected RNA processing genes. These observations collectively indicate that the CoREST complex and MYC cooperate to transcriptionally activate a subset of genes involved in RNA processing in melanoma cells.

### 3.3. NOLC1 Is a Downstream Target of CoREST Complex and Associated with Poor Prognosis in Melanoma Patients

**Figure 3:**
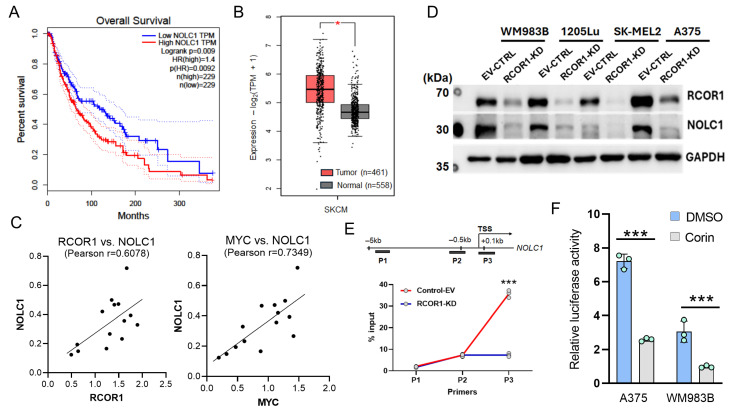
NOLC1 is highly expressed in cancer patients and is transcriptionally regulated by the CoREST complex. (**A**) Kaplan–Meier’s survival curves of melanoma patients with high and low expressions of NOLC1 gene transcript levels, defined by median expression across patients (TCGA SKCM data set). (**B**) A box plot analysis comparing the expression level of *NOLC1* gene between melanoma tissues from patients (TCGA data set) and normal skin tissues from donors (GTEx data set). (**C**) Positive correlations in expression levels of NOLC1 with RCOR1 and MYC proteins in a panel of 14 melanoma cell lines (n = 14). (**D**) Western blot analysis showing that RCOR1 KD decreases the protein level of NOLC1 in a panel of melanoma cell lines. (**E**) ChIP qPCR of the active histone mark H3K27Ac at the promoter regions of NOLC1. The upper panel is a schematic of the PCR primer locations. The enrichment of H3K27Ac was significantly decreased at the TSS of the NOLC1 regulatory region with the knockdown of RCOR1. Error bars represent geomean ±SD of three biological replicates and s two-sided Student’s *t*-test was used: *** *p* < 0.001. (**F**) Promoter–reporter assays of the *NOLC1* gene promoter with and without Corin treatment in two melanoma cell lines, WM983B and A375, compared with DMSO control. Error bars represent geomean ± SD of three biological replicates and two-sided Student’s *t*-test was used: *** *p* < 0.001.

The expression levels of RCOR1 show a significant correlation with a subset of RNA processing genes regulated by the CoREST complex in cancer patients, including melanoma (Figure S4). Among the 62 genes (Figure 2C) whose expression was controlled by both the CoREST complex and MYC, 22 genes showed a statistically significant positive correlation with *RCOR1* expression, while only 8 genes were inversely correlated in melanoma patient samples from The Cancer Genome Atlas (TCGA SKCM data set) [34] (Figure S4A). A very similar pattern was observed in the TCGA pan-cancer data set, where *NOLC1* was one of the top two genes positively correlated with *RCOR1* expression (Figure S4B). Amongst these 22 genes, *NOLC1*, encoding nucleolar coiled-body phosphoprotein 1 (NOPP140), showed significantly poor survival in melanoma patients with high expression compared with those with low expression (Figure 3A).

We also compared the *NOLC1* expression in human cutaneous melanomas (TCGA data set) with normal skin tissue from the Genotype-Tissue Expression (GTEx) project [26]. While this comparison was not ideal because melanocytes are simply one component of normal skin, the expression level in skin provides a stable baseline for the comparison. We found that *NOLC1* transcript levels are significantly higher in melanoma tissues compared with normal skin (Figure 3B). We then compared the protein level of NOLC1 with RCOR1 and MYC in a panel of fourteen melanoma cell lines. There was a direct co-relation between NOLC1 protein levels and those of both RCOR1 and MYC (Figure 3C).

To further investigate the regulation of NOLC1 by the CoREST complex, we depleted RCOR1 by shRNA in four melanoma cell lines. The protein level of NOLC1 was significantly decreased upon RCOR1 depletion (Figure 3D). Furthermore, a ChIP-qPCR assay of the active histone mark H3K27Ac at the transcription start site (TSS) of *NOLC1* confirmed an active chromatin state in the control cells, while there was a significant decrease in the RCOR1-KD cells (Figure 3E). To further corroborate the transcriptional regulatory role of the CoREST complex on *NOLC1*, we cloned the promoter of *NOLC1* in the pGL4.10 plasmid and performed a promoter–reporter assay in two melanoma cell lines, A375 and WM983B, with and without the CoREST complex inhibitor, Corin. The promoter activity of *NOLC1* was significantly decreased by Corin treatment (Figure 3F). Taken together, these observations suggest that NOLC1 is a transcriptional target co-regulated by the CoREST complex and MYC, and that its overexpression may play a significant role in melanoma progression.

### 3.4. NOLC1 Regulates the U1 snRNA Nm and Alternative Splicing and Promotes Melanoma Cell Viability

NOLC1 is localized in the nucleolus and Cajal bodies and plays a key role in the localization and function of small nucleolar ribonucleoproteins (snoRNPs) and small Cajal body-specific ribonucleoproteins (scaRNPs) [35]. SnoRNAs, essential components of snoRNPs, are non-coding RNAs that guide the chemical modification of rRNAs and transfer RNAs (tRNAs) by either Nm or the isomerization of uridines to pseudouridines. ScaRNPs catabolize the Nm of snRNAs including spliceosomal small nuclear RNAs which are crucial for high-fidelity spliceosome formation. These modifications can affect the expression and function of these RNAs and associated proteins [36]. Specifically, spliceosomal small nuclear RNAs (snRNAs) are the core members of nuclear splicing machinery, which is vital for diversifying the protein-coding potential of the genome [37]. The major snRNAs, U1, U2, U4, and U6, play a key role in splicing, and can also be modified by Nm or pseudouridination [38].

Previously, Nm and pseudouridylation were reported in human U1 snRNA, specifically 2′-O-ribose methylation at residue A70 (Am70) [38,39]. To investigate a potential connection between the CoREST-NOLC1 pathway and the modification of snRNAs, we used U1 snRNA as a surrogate readout for splicing changes in melanoma cells. The Am70 modification is known to inhibit reverse transcriptase activity at low dNTP concentrations due to steric hindrance or a conformational change [40]. Avian Myeloblastosis Virus (AMV) reverse transcriptase is the enzyme of choice for mapping Nm sites in RNA templates because of its sensitivity to these modifications [41].

**Figure 4:**
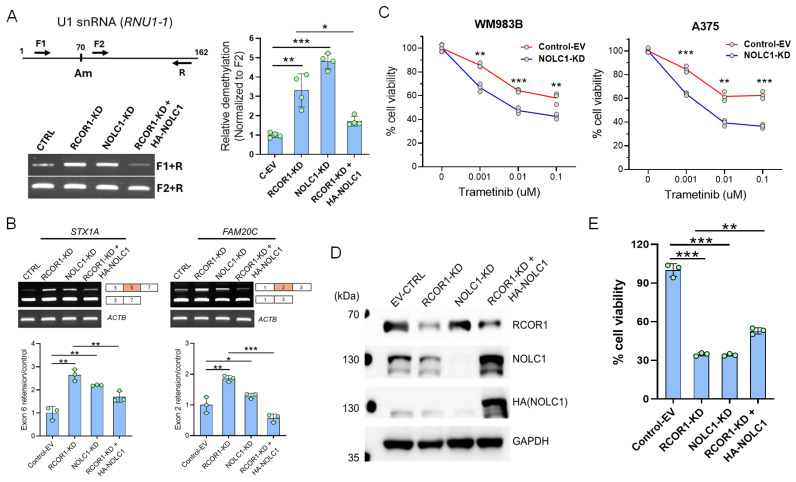
NOLC1 is a downstream effector of the CoREST complex and plays a significant role in mRNA splicing via snRNA Nm modification and cell viability in melanoma. (**A**) Schematic diagram of the PCR primers of RTL-P (reverse transcription at low deoxy-ribonucleoside triphosphate (dNTP) concentrations followed by PCR) assay for Nm modification. F1 is upstream of the Nm residue at A70 and F2 is downstream of the Nm residue. A representative assay of RTL-P analysis shows the increased amplification from U1 snRNA (*RNU1-1*) isolated from RCOR1- and NOLC1-KD cells, and low amplification in the ectopically expressed NOLC1 in the RCOR1-KD cells with F1+R primers. The F2+R primers had no difference in the PCR amplification used as a control (left panel). Quantification of Nm levels by RTL-P analysis of the *RNU1-1* shows a significant increase in RTL-P amplification when in the RCOR1- and NOLC1-KD cells and strong decrease in RTP-L amplification when NOLC1 was ectopically expressed in the RCOR1 knockdown cells compared with a control empty vector. Error bars represent geomean ± SD of three biological replicates and a two-sided Student’s *t*-test was used: * *p* < 0.05, ** *p* < 0.01, and *** *p* < 0.001 (right panel). (**B**) Semi-quantitative RT-PCR showing the retentions of exon 6 in *STX1A* and exon 2 in *FAM20C* gene transcripts isolated from the RCOR1- and NOLC1-KD cells, respectively. These retentions were decreased when NOLC1 was ectopically expressed in the RCOR1-KD cells. The upper panel shows the agarose gel images, and the lower panel shows the ImageJ (version 1.54p) quantification and normalization with the control of 18srRNA. Error bars represent geomean ±SD of three biological replicates and a two-sided Student’s *t*-test was used: * *p* < 0.05, ** *p* < 0.01, and *** *p* < 0.001. (**C**) Cell viability assay of WM983B and A375 melanoma cell lines with NOLC1-KD and isogenic control cells with increasing concentrations of MEK inhibitor trametinib. Error bars represent geomean ±SD of three biological replicates and a two-sided Student’s *t*-test was used: ** *p* < 0.01, and *** *p* < 0.001. (**D**) Representative Western blot from duplicate experiments, showing the knockdown of RCOR1 and NOLC1 and ectopic expression of HA-tagged NOLC1 (HA-NOLC1) in the A375 cell line. (**E**) Cell viability assay with CellTiter-Glo 2.0 reagent. The cell viability was strongly decreased with the RCOR1 and NOLC1 knockdown but partially recovered when NOLC1 was expressed in the RCOR1-KD cells. Error bars represent geomean ± SD of three biological replicates and a two-sided Student’s *t*-test was used: ** *p* < 0.01, and *** *p* < 0.001.

To investigate the potential Am70 modification in U1 snRNA, we knocked down RCOR1 and NOLC1 using shRNA and ectopically expressed NOLC1 in the RCOR1 knockdown cells (Figure 4D). We designed a pair of forward primers (F1 upstream of the Am70 residue and F2 downstream) and a single reverse primer for a quantitative PCR (qPCR) reaction. First-strand cDNAs were synthesized with low dNTP concentrations using AMV reverse transcriptase as previously described [41]. Interestingly, the amplification of the U1 snRNA transcript using the F1 primer was increased in the RCOR1- and NOLC1-depleted cells. This observation indicates that the Am70 modification, which normally stalls the reverse transcriptase, was erased with the downregulation of RCOR1 and NOLC1. Conversely, the ectopic expression of NOLC1 in the RCOR1 knockdown cells decreased the amplification of the U1 snRNA transcript, implying that the Am70 modification was restored to control levels (Figure 4A). There was no change in the amplification using the F2 primer, which is located downstream of the Am70 residue. This result suggests that NOLC1, in a manner dependent on RCOR1, methylates the A70 residue of U1 snRNA.

To further corroborate the molecular connection of RCOR1/NOLC1-induced U1 snRNA Nm modification to the alternative splicing switching observed from the RNA-seq data (Figure 1C and Figure S1B), we selected two genes (*STX1A* and *FAM20C*) identified for their differential exon usage in RCOR1-KD A375 cells. We experimentally verified their isoform switching using freshly prepared mRNA samples. For example, we observed that exon 6 of *STX1A* was retained in both RCOR1-KD and NOLC1-KD cells compared with the control cells. Interestingly, this exon retention was reversed when NOLC1 was ectopically expressed in the RCOR1-KD A375 cells (Figure 4B). Similarly, the *FAM20C* gene had exon retention between exon 1 and 3 in RCOR1 and NOLC1 knockdown cells. This retention was also reversed when NOLC1 was ectopically expressed in the RCOR1-KD cells (Figure 4B). These observations strongly suggest that the CoREST complex plays a role in alternative splicing by regulating the RNA processing genes including NOLC1.

Previous studies have shown that the expression of NOLC1 increases in non-small cell lung cancer, promoting multidrug resistance (MDR), and that its downregulation sensitizes MDR cells [42]. To explore NOLC1’s potential role in the sensitization of melanoma cells to MEK inhibitors (MEKis), we downregulated NOLC1 by shRNA and treated two cell lines, A375 and WM983B, with increasing concentrations of trametinib, a clinically approved drug for advanced melanoma treatment. Both the cell lines showed increased sensitivity to trametinib with NOLC1 knockdown compared with the control (Figure 4C). To further probe the role of NOLC1 in the context of RCOR1-KD cells, we depleted the RCOR1 and NOLC1 by shRNAs and ectopically expressed the HA-tagged NOLC1 (HA-NOLC1) in the RCOR-KD A375 cell line (Figure 4D). The downregulation of RCOR1 and NOLC1 significantly decreased cell viability. However, a modest but statistically significant recovery in cell viability was observed when NOLC1 was ectopically expressed in the RCOR1-KD cells (Figure 4E). This result mirrors the observed effects on U1 snRNA methylation and alternative splicing, highlighting a consistent pathway. These observations suggest that RCOR1 regulates the 2′-O-ribose methylation (Nm) of snRNAs through NOLC1, which in turn affects the alternative splicing of mRNA and ultimately influences cell survival.

## 4. Discussion

In this study, we have identified that the CoREST complex transcriptionally upregulates the expression of NOLC1. The CoREST complex is primarily known for its transcription repression function through HDAC1/2 and LSD1 by erasing the active histone marks; however, it has also been detected in the regulatory regions of active genes [6]. Depending on its interacting partner protein, the CoREST complex can also promote gene expression [43]. For the regulation of NOLC1, the CoREST complex may exploit the gene-activating role of MYC, as both are part of the same complex (our unpublished data, manuscript in review). We also detected the binding of both the CoREST complex and MYC in the regulatory regions of RNA processing genes (Figure 2A), and both RCOR1 and MYC can be detected by ChIP-Re-ChIP assay at the promoters of these genes, indicating that they are part of the same complex, at least in the regulatory regions of these genes examined (Figure 2E).

Melanoma is a type of cancer in which tumor biology, gene expression, treatment responses, and patient outcomes can differ by sex [44,45]. To mitigate these issues, this study included melanoma cell lines derived from both female (A375) and male (WM983B, SK-MEL2) patients. RNA-sequencing following RCOR1 KD revealed a substantial overlap in the differential expression profiles of RNA processing genes, including NOLC1, across these cell lines regardless of sex. Furthermore, the CoREST complex-dependent regulation of NOLC1 expression and the resulting reduction in cell viability upon NOLC1 knockdown combined with an MEK inhibitor were consistently observed in cell lines from both sexes (Figure 3D and Figure 4C). These findings suggest that the CoREST–MYC regulatory axis functions similarly in melanoma cells independent of sex origin, presuming that no significant sex bias was introduced in this study.

The regulation of RNA processing genes by CoREST-MYC is significant because the cancer cells use the alternative splicing strategy to produce flexible proteomes that support survival and growth [46]. Approximately 50% of genetic diseases in humans are caused by genetic mutations in splice site sequences and regulatory elements, resulting in alternative exon patterns [47,48,49]. Changes in the splicing pattern of cancer-related genes could affect various signaling pathways, cell proliferation, apoptosis, angiogenesis, and metastasis [50,51]. In TCGA data sets, we observed that NOLC1 was among the top genes correlated with RCOR1 expression both in pan-cancer and melanoma patient cohorts (Supplementary Figure S4). Notably, melanoma patients with high NOLC1 expression showed poor survival outcomes (Figure 3A).

**Figure 5:**
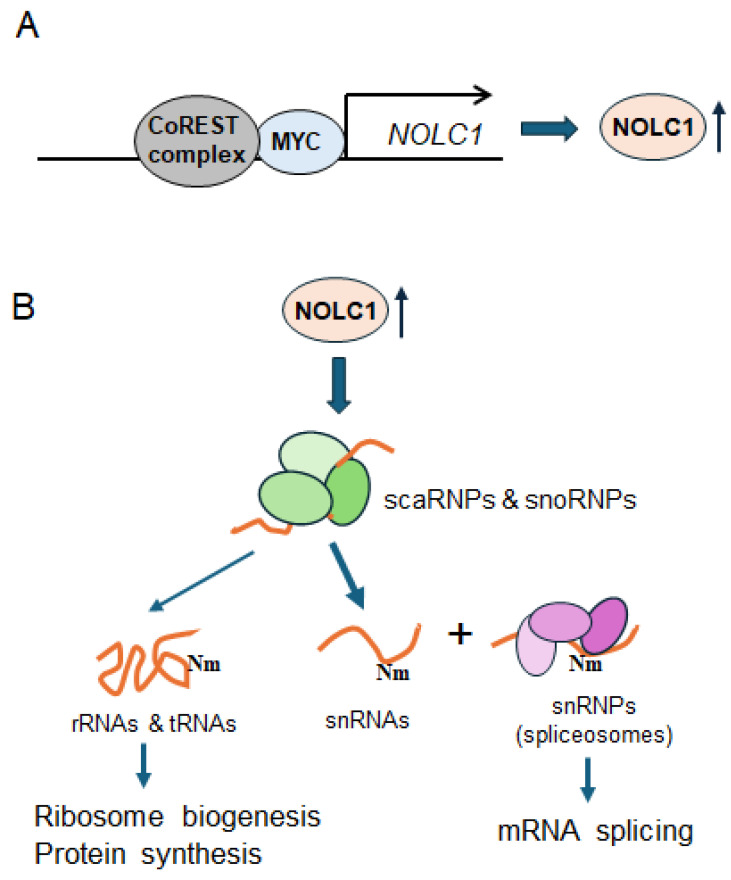
A schematic diagram depicting the CoREST complex modulating RNA splicing via NOLC1 in melanoma cells. (**A**) The CoREST complex transcriptionally upregulates *NOLC1* and other genes associated with RNA processing in coordination with MYC. (**B**) The elevated NOLC1 protein ensures the proper maturation, assembly, and localization of snoRNPs and scaRNPs within the nucleolus and Cajal bodies. Then, scaRNPs carry out site-specific Nm in snRNAs including spliceosomal snRNAs, while snoRNPs primary target rRNAs and tRNAs for Nm modifications. These site-specific Nm modifications are critical for ribosome biogenesis, protein synthesis, and mRNA splicing.

NOLC1 is a critical chaperone for the biogenesis and assembly of small nucleolar ribonucleoproteins (snoRNPs) and small Cajal body-specific ribonucleoproteins (scaRNPs). SnoRNPs and scaRNPs are protein-RNA complexes that guide post-transcriptional modifications on other RNA species, including snRNA Nm [52]. Based on the experimental evidence presented in this study, a detailed molecular pathway can be potentially constructed that explains how the CoREST complex, through the MYC oncoprotein and NOLC1 chaperone, regulates mRNA splicing. This pathway provides a plausible mechanism for the observed splicing variants upon RCOR1 KD and unites the disparate functions of these proteins (Figure 5). First, in coordination with MYC, the CoREST complex drives the expression of genes encoding post-transcriptional RNA processing factors, including the gene encoding the chaperone protein NOLC1. The increase in NOLC1 protein leads to a higher concentration of this chaperone, which is then available to ensure the proper maturation, assembly, and localization of snoRNPs and scaRNPs within the nucleolus and Cajal bodies. These mature and correctly localized snRNPs are essential for guiding the site-specific Nm of various snRNAs. The Nm modification is particularly relevant for spliceosomal snRNAs like U1, which are central components of the spliceosome. Nm modification on snRNAs stabilizes their structure and ensures the optimal fidelity of catalytic activity of the spliceosome. When the CoREST complex is inhibited (e.g., by RCOR1 KD), NOLC1 protein level declines. This compromises snoRNP maturation and localization, resulting in a loss of Nm modification on snRNAs, and consequent widespread alternative splicing variants observed in melanoma cells. A critical piece of evidence supporting this hypothesis is the finding that the ectopic expression of NOLC1 in RCOR1-depleted cells can restore Nm modification (Figure 4A) and alternative splicing variant formation (Figure 4B).

While the CoREST-MYC-NOLC1 pathway provides a robust explanation for a significant portion of the observed splicing changes, it is important to acknowledge that this is likely not the only regulatory mechanism at play. The CoREST complex appears to employ a multi-layered regulatory strategy to control alternative splicing. In addition to transcriptionally controlling the expression of RNA processing genes like *NOLC1*, the complex has been shown to physically and directly interact with a diverse array of RNA splicing factors [53]. The pharmacological inhibition of the CoREST complex, for example, with the small molecule inhibitor, Corin, disrupts these direct protein–protein interactions with splicing factors [53]. This suggests that the CoREST complex influences splicing through at least two independent mechanisms: an indirect, transcriptional one via the CoREST-MYC-NOLC1 axis, and a direct, physical one that modulates the function of the splicing machinery itself. This dual-pronged control may provide a deeper understanding of why the disruption of the CoREST complex leads to such extensive and widespread changes in the splicing landscape.

The dysregulation of mRNA splicing is a key driver of cancer, enabling tumors to generate protein isoforms that enhance proliferation, survival, and immune evasion [54]. The disruption of the CoREST-MYC-NOLC1 pathway actively promotes these aberrant splicing events, which not only suppress tumor growth but also produce novel, immunogenic splice-neoantigens capable of triggering anti-tumor immune responses [55,56,57]. This dual effect highlights the therapeutic potential of targeting the CoREST-NOLC1 axis: inhibiting this pathway both blocks a pro-tumor mechanism and generates tumor-specific antigens that may sensitize “immune-cold” cancers to immunotherapies like checkpoint blockade, warranting follow-up in vivo studies to evaluate the role of the CoREST complex in regulating RNA splicing and shaping tumor immunogenicity. Such studies will be necessary to determine whether the effects of CoREST inhibition translate into measurable anti-tumor efficacy in animal models. These in vivo investigations are currently underway.

## 5. Conclusions

In summary, our study uncovers a previously unrecognized transcriptional mechanism by which the CoREST complex, in cooperation with MYC, regulates RNA splicing via the downstream effector NOLC1. The CoREST-driven expression of NOLC1 regulates the snoRNP-dependent Nm of U1 snRNA, ensuring spliceosome integrity and proper mRNA processing in melanoma cells. The disruption of this axis induces widespread splicing isoform expression, linking chromatin regulation to RNA processing. These findings establish the CoREST–MYC–NOLC1 pathway as a critical integrator of transcriptional and post-transcriptional control with therapeutic potential in CoREST-dysregulated cancers.

## Data Availability

Raw RNA-seq and ChIP-seq files have been deposited in the National Center for Biotechnology Information BioProject (access code: PRJNA996569). ChIP sequencing data for K562 (access codes: 84245 (H3K27Ac), 45560 (MYC), 01906 (RCOR1)) and MCF7 (access codes: 8183 (MYC), 63457 (RCOR1)) cell lines are available from Cistrome DB (http://cistrome.org/). All other raw data are available upon request from the corresponding author.

## Supplementary Materials

The following supporting information can be downloaded at: https://www.mdpi.com/article/10.3390/cells14211699/s1, Figure S1: Heatmaps showing genes predominantly upregulated by RCOR1 knockdown (RCOR1-KD) in three melanoma cell lines. (A) Top 15 genes from human GOMF_CYTOKINE_ACTIVITY gene set (GO:0005125) upregulated by RCOR1-KD in A375, WM983B, and SK-MEL2 melanoma cells. (B) Top 15 genes from human GOBP_PATTERN_RECOGNITION_RECEPTOR_SIGNALING_PATHWAY gene set (GO:0062207) that are upregulated by RCOR1-KD in these melanoma cell lines; Figure S2. RNA processing genes are down regulated by CoREST complex inhibitor Corin. (A) RT-PCR showing the downregulation of RNA processing genes following Corin treatment in A375, WM983B, and SK-MEL2 cell lines. (B) Splicing analysis of significantly altered genes using IsoformSwitchAnalyzer on the RNAseq data comparing Corin-treated cells with DMSO controls. Alternative splicing events are classified as A3 (alternative 3’ splice site), A5 (alternative 5’ splice site), ATSS (alternative transcription start site), ATTS (alternative transcription termination site), ES (exon skipping), IR (intron retention), MEE (mutually exclusive exons), and MES (mutually exclusive splicing/exons); Figure S3. Direct transcriptional regulation of RNA processing genes by the CoREST complex and MYC in a melanoma cell line (A375). (A) ChIP-seq peaks of the RCOR1 and MYC binding (top lanes) and RNA-seq (bottom lanes) at a group of selected gene promoters annotated with RNA processing (GOBP_RNA_PROCESSING, GO:0006396). (B) The RCOR1 and MYC binding was confirmed by ChIP qPCR at a group of selected genes; Figure S4: Expression correlation of RCOR1 with and RNA processing genes in patient samples. Spearman correlation coefficient (ρ) with statistical significances (q-value < 0.001) between the expression of *RCOR1* and each of the 62 genes annotated as RNA processing and occupied by both RCOR1 and MYC in the promoter region. (A) Patients with cutaneous melanoma (SKCM). (B) All cancer types (Pan-cancer). TCGA datasets were used; Table S1: Antibodies and primer sequences for RT-PCR, RTL-P, ChIP, and cloning for reporter vector.

## Abbreviations

NOLC1/NOPP140	Nucleolar coiled-body phosphoprotein 1
snRNP	Small nuclear ribonucleoproteins
LSD1	Lysine-specific histone demethylase
REST	RE1 Silencing Transcription factor
NRSF	Neural-Restrictive Silencer Factor
ChIP	Chromatin immunoprecipitation
RTL-P	Reverse Transcription at Low deoxy-ribonucleoside triphosphate (dNTP) concentrations followed by polymerase chain reaction (PCR)
GSEA	Gene Set Enrichment Analysis
ChIP-seq	Chromatin immunoprecipitation sequencing
GO	Gene Ontology
GOMo	Gene Ontology for Motifs
TCGA	The Cancer Genome Atlas
GTEx	Genome-Tissue Expression
TSS	Transcription start site
snoRNPs	Small nucleolar ribonucleoproteins
scaRNPs	Small Cajal body-specific ribonucleoproteins
tRNA	Transfer RNA
rRNA	Ribosomal RNA
snRNA	Small nuclear RNA
qPCR	Quantitative PCR
MDR	Multidrug resistance
shRNA	Short hairpin RNA
HDAC	Histone deacetylase

## References

[1] Ballas N., Battaglioli E., Atouf F., Andres M.E., Chenoweth J., Anderson M.E., Burger C., Moniwa M., Davie J.R., Bowers W.J. (2001). Regulation of neuronal traits by a novel transcriptional complex. Neuron.

[2] Humphrey G.W., Wang Y., Russanova V.R., Hirai T., Qin J., Nakatani Y., Howard B.H. (2001). Stable histone deacetylase complexes distinguished by the presence of SANT domain proteins CoREST/kiaa0071 and Mta-L1. J. Biol. Chem..

[3] You A., Tong J.K., Grozinger C.M., Schreiber S.L. (2001). CoREST is an integral component of the CoREST- human histone deacetylase complex. Proc. Natl. Acad. Sci. USA.

[4] Shi Y., Lan F., Matson C., Mulligan P., Whetstine J.R., Cole P.A., Casero R.A., Shi Y. (2004). Histone demethylation mediated by the nuclear amine oxidase homolog LSD1. Cell.

[5] Andres M.E., Burger C., Peral-Rubio M.J., Battaglioli E., Anderson M.E., Grimes J., Dallman J., Ballas N., Mandel G. (1999). CoREST: A functional corepressor required for regulation of neural-specific gene expression. Proc. Natl. Acad. Sci. USA.

[6] Rivera C., Lee H.G., Lappala A., Wang D., Noches V., Olivares-Costa M., Sjoberg-Herrera M., Lee J.T., Andres M.E. (2022). Unveiling RCOR1 as a rheostat at transcriptionally permissive chromatin. Nat. Commun..

[7] Bennesch M.A., Segala G., Wider D., Picard D. (2016). LSD1 engages a corepressor complex for the activation of the estrogen receptor alpha by estrogen and cAMP. Nucleic Acids Res..

[8] Metzger E., Wissmann M., Yin N., Muller J.M., Schneider R., Peters A.H., Gunther T., Buettner R., Schule R. (2005). LSD1 demethylates repressive histone marks to promote androgen-receptor-dependent transcription. Nature.

[9] Wang J., Scully K., Zhu X., Cai L., Zhang J., Prefontaine G.G., Krones A., Ohgi K.A., Zhu P., Garcia-Bassets I. (2007). Opposing LSD1 complexes function in developmental gene activation and repression programmes. Nature.

[10] Choquet K., Patop I.L., Churchman L.S. (2025). The regulation and function of post-transcriptional RNA splicing. Nat. Rev. Genet..

[11] Alfonso-Gonzalez C., Hilgers V. (2024). (Alternative) transcription start sites as regulators of RNA processing. Trends Cell Biol..

[12] Nott A., Le Hir H., Moore M.J. (2004). Splicing enhances translation in mammalian cells: An additional function of the exon junction complex. Genes Dev..

[13] Bonnal S.C., Lopez-Oreja I., Valcarcel J. (2020). Roles and mechanisms of alternative splicing in cancer—Implications for care. Nat. Rev. Clin. Oncol..

[14] Bradley R.K., Anczukow O. (2023). RNA splicing dysregulation and the hallmarks of cancer. Nat. Rev. Cancer.

[15] Cai W., Xiao C., Fan T., Deng Z., Wang D., Liu Y., Li C., He J. (2024). Targeting LSD1 in cancer: Molecular elucidation and recent advances. Cancer Lett..

[16] Li M., Dai M., Cheng B., Li S., Guo E., Fu J., Ma T., Yu B. (2024). Strategies that regulate LSD1 for novel therapeutics. Acta Pharm. Sin. B.

[17] Zheng R., Pan Y., Liu X., Liu F., Li A., Zheng D., Luo Y. (2023). Comprehensive analysis of REST corepressors (RCORs) in pan-cancer. Front. Cell Dev. Biol..

[18] Min D., Byun J., Lee E.-J., Khan A.A., Liu C., Loudig O., Hu W., Zhao Y., Herlyn M., Tycko B. (2022). Epigenetic Silencing of BMP6 by the SIN3A–HDAC1/2 Repressor Complex Drives Melanoma Metastasis via FAM83G/PAWS1. Mol. Cancer Res..

[19] Anders S., Reyes A., Huber W. (2012). Detecting differential usage of exons from RNA-seq data. Genome Res..

[20] Love M.I., Huber W., Anders S. (2014). Moderated estimation of fold change and dispersion for RNA-seq data with DESeq2. Genome Biol..

[21] Khan A.A., Ham S.J., Yen L.N., Lee H.L., Huh J., Jeon H., Kim M.H., Roh T.Y. (2018). A novel role of metal response element binding transcription factor 2 at the Hox gene cluster in the regulation of H3K27me3 by polycomb repressive complex 2. Oncotarget.

[22] Zhang Y., Liu T., Meyer C.A., Eeckhoute J., Johnson D.S., Bernstein B.E., Nusbaum C., Myers R.M., Brown M., Li W. (2008). Model-based analysis of ChIP-Seq (MACS). Genome Biol..

[23] Khan A., Mathelier A. (2017). Intervene: A tool for intersection and visualization of multiple gene or genomic region sets. BMC Bioinform..

[24] Mi H., Lazareva-Ulitsky B., Loo R., Kejariwal A., Vandergriff J., Rabkin S., Guo N., Muruganujan A., Doremieux O., Campbell M.J. (2005). The PANTHER database of protein families, subfamilies, functions and pathways. Nucleic Acids Res..

[25] Dong Z.-W., Shao P., Diao L.-T., Zhou H., Yu C.-H., Qu L.-H. (2012). RTL-P: A sensitive approach for detecting sites of 2′-O-methylation in RNA molecules. Nucleic Acids Res..

[26] Consortium G.T. (2020). The GTEx Consortium atlas of genetic regulatory effects across human tissues. Science.

[27] Tang Z., Kang B., Li C., Chen T., Zhang Z. (2019). GEPIA2: An enhanced web server for large-scale expression profiling and interactive analysis. Nucleic Acids Res..

[28] Abrajano J.J., Qureshi I.A., Gokhan S., Molero A.E., Zheng D., Bergman A., Mehler M.F. (2010). Corepressor for element-1-silencing transcription factor preferentially mediates gene networks underlying neural stem cell fate decisions. Proc. Natl. Acad. Sci. USA.

[29] Qureshi I.A., Gokhan S., Mehler M.F. (2010). REST and CoREST are transcriptional and epigenetic regulators of seminal neural fate decisions. Cell Cycle.

[30] Abrajano J.J., Qureshi I.A., Gokhan S., Zheng D., Bergman A., Mehler M.F. (2009). REST and CoREST modulate neuronal subtype specification, maturation and maintenance. PLoS ONE.

[31] Yu H.-B., Johnson R., Kunarso G., Stanton L.W. (2011). Coassembly of REST and its cofactors at sites of gene repression in embryonic stem cells. Genome Res..

[32] Gómez A.V., Galleguillos D., Maass J.C., Battaglioli E., Kukuljan M., Andrés M.E. (2008). CoREST represses the heat shock response mediated by HSF1. Mol. Cell.

[33] Buske F.A., Boden M., Bauer D.C., Bailey T.L. (2010). Assigning roles to DNA regulatory motifs using comparative genomics. Bioinformatics.

[34] Cancer Genome Atlas Research N., Weinstein J.N., Collisson E.A., Mills G.B., Shaw K.R., Ozenberger B.A., Ellrott K., Shmulevich I., Sander C., Stuart J.M. (2013). The Cancer Genome Atlas Pan-Cancer analysis project. Nat. Genet..

[35] Bizarro J., Deryusheva S., Wacheul L., Gupta V., Ernst F.G., Lafontaine D.L., Gall J.G., Meier U.T. (2021). Nopp140-chaperoned 2′-O-methylation of small nuclear RNAs in Cajal bodies ensures splicing fidelity. Genes Dev..

[36] Elliott B.A., Ho H.-T., Ranganathan S.V., Vangaveti S., Ilkayeva O., Abou Assi H., Choi A.K., Agris P.F., Holley C.L. (2019). Modification of messenger RNA by 2′-O-methylation regulates gene expression in vivo. Nat. Commun..

[37] Wilkinson M.E., Charenton C., Nagai K. (2020). RNA splicing by the spliceosome. Annu. Rev. Biochem..

[38] Morais P., Adachi H., Yu Y.-T. (2021). Spliceosomal snRNA epitranscriptomics. Front. Genet..

[39] Darzacq X., Jády B.E., Verheggen C., Kiss A.M., Bertrand E., Kiss T. (2002). Cajal body-specific small nuclear RNAs: A novel class of 2′-O-methylation and pseudouridylation guide RNAs. EMBO J..

[40] Decombe A., Peersen O., Sutto-Ortiz P., Chamontin C., Piorkowski G., Canard B., Nisole S., Decroly E. (2024). Internal RNA 2′-O-methylation on the HIV-1 genome impairs reverse transcription. Nucleic Acids Res..

[41] Maden B.E.H. (2001). Mapping 2′-O-methyl groups in ribosomal RNA. Methods.

[42] Huang H., Li T., Chen M., Liu F., Wu H., Wang J., Chen J., Li X. (2018). Identification and validation of NOLC1 as a potential target for enhancing sensitivity in multidrug resistant non-small cell lung cancer cells. Cell. Mol. Biol. Lett..

[43] Zeng C., Chen J., Cooke E.W., Subuddhi A., Roodman E.T., Chen F.X., Cao K. (2023). Demethylase-independent roles of LSD1 in regulating enhancers and cell fate transition. Nat. Commun..

[44] Murali T., Schwartz M., Reynolds A.Z., Luo L., Ridgeway G., Busam K.J., Cust A.E., Anton-Culver H., Gallagher R.P., Zanetti R. (2025). Sex differences in melanoma survival-a GEM study. JNCI Cancer Spectr..

[45] Schadendorf D., van Akkooi A.C.J., Berking C., Griewank K.G., Gutzmer R., Hauschild A., Stang A., Roesch A., Ugurel S. (2018). Melanoma. Lancet.

[46] Kim Y.-J., Kim H.-S. (2012). Alternative splicing and its impact as a cancer diagnostic marker. Genom. Inform..

[47] Matlin A.J., Clark F., Smith C.W. (2005). Understanding alternative splicing: Towards a cellular code. Nat. Rev. Mol. Cell Biol..

[48] Cáceres J.F., Kornblihtt A.R. (2002). Alternative splicing: Multiple control mechanisms and involvement in human disease. TRENDS Genet..

[49] Cartegni L., Chew S.L., Krainer A.R. (2002). Listening to silence and understanding nonsense: Exonic mutations that affect splicing. Nat. Rev. Genet..

[50] Skotheim R.I., Nees M. (2007). Alternative splicing in cancer: Noise, functional, or systematic?. Int. J. Biochem. Cell Biol..

[51] Venables J.P. (2006). Unbalanced alternative splicing and its significance in cancer. BioEssays News Rev. Mol. Cell Dev. Biol..

[52] Zhai F., Wang J., Luo X., Ye M., Jin X. (2023). Roles of NOLC1 in cancers and viral infection. J. Cancer Res. Clin. Oncol..

[53] Fisher R.J., Park K., Lee K., Pinjusic K., Vanasse A., Ennis C.S., Ficcaro S., Marto J., Stransky S., Duke-Cohan J. (2024). CoREST Complex Inhibition Alters RNA Splicing to Promote Neoantigen Expression and Enhance Tumor Immunity. bioRxiv.

[54] Zhu S., Zhang T., Zheng L., Liu H., Song W., Liu D., Li Z., Pan C.-X. (2021). Combination strategies to maximize the benefits of cancer immunotherapy. J. Hematol. Oncol..

[55] Minati R., Perreault C., Thibault P. (2020). A roadmap toward the definition of actionable tumor-specific antigens. Front. Immunol..

[56] Jou J., Harrington K.J., Zocca M.-B., Ehrnrooth E., Cohen E.E. (2021). The changing landscape of therapeutic cancer vaccines—Novel platforms and neoantigen identification. Clin. Cancer Res..

[57] Liu J., Fu M., Wang M., Wan D., Wei Y., Wei X. (2022). Cancer vaccines as promising immuno-therapeutics: Platforms and current progress. J. Hematol. Oncol..

