# Supplementary material for: The CoREST Complex Regulates Alternative Splicing by the Transcriptional Regulation of RNA Processing Genes in Melanoma Cells
Source: Cells. 2025 Oct 29;14(21):1699. doi: 10.3390/cells14211699 (PMC12607843; doi:10.3390/cells14211699)
Supplement: Supplementary file 1 [file cells-14-01699-s001.zip › cells-3866217-supplementary.pdf]

cells

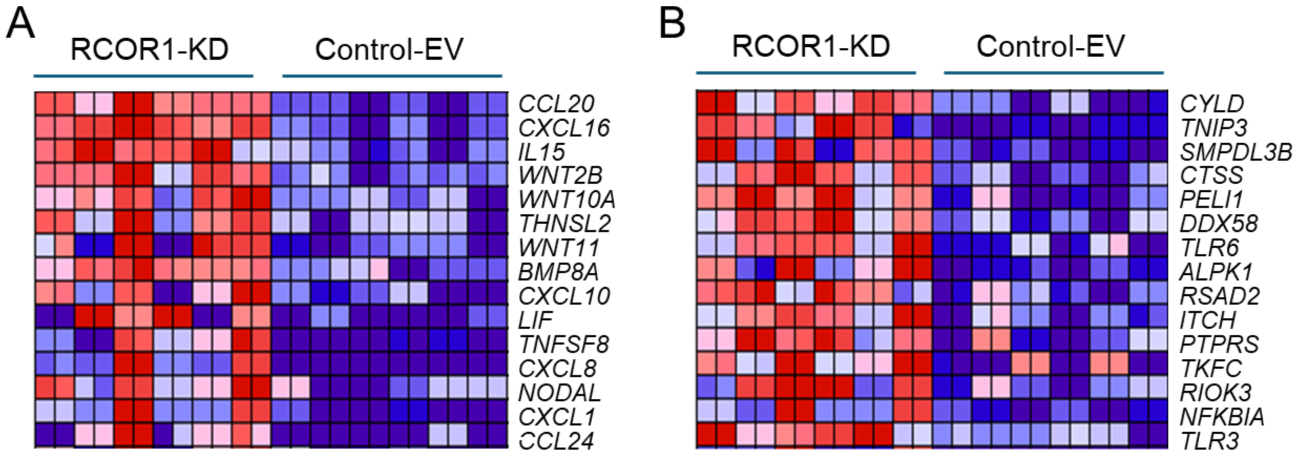

Figure S1. Heatmaps showing genes predominantly upregulated by RCOR1 knockdown (RCOR1-KD) in three melanoma cell lines. A) Top 15 genes from human GOMF_CYTOKINE_ACTIVITY gene set (GO:0005125) upregulated by RCOR1-KD in A375, WM983B, and SK-MEL2 melanoma cells. **B)** Top 15 genes from human GOBP_PATTERN_RECOGNITION_RECEPTOR_SIGNALING_PATHWAY gene set (GO:0062207) that are upregulated by RCOR1-KD in these melanoma cell lines.

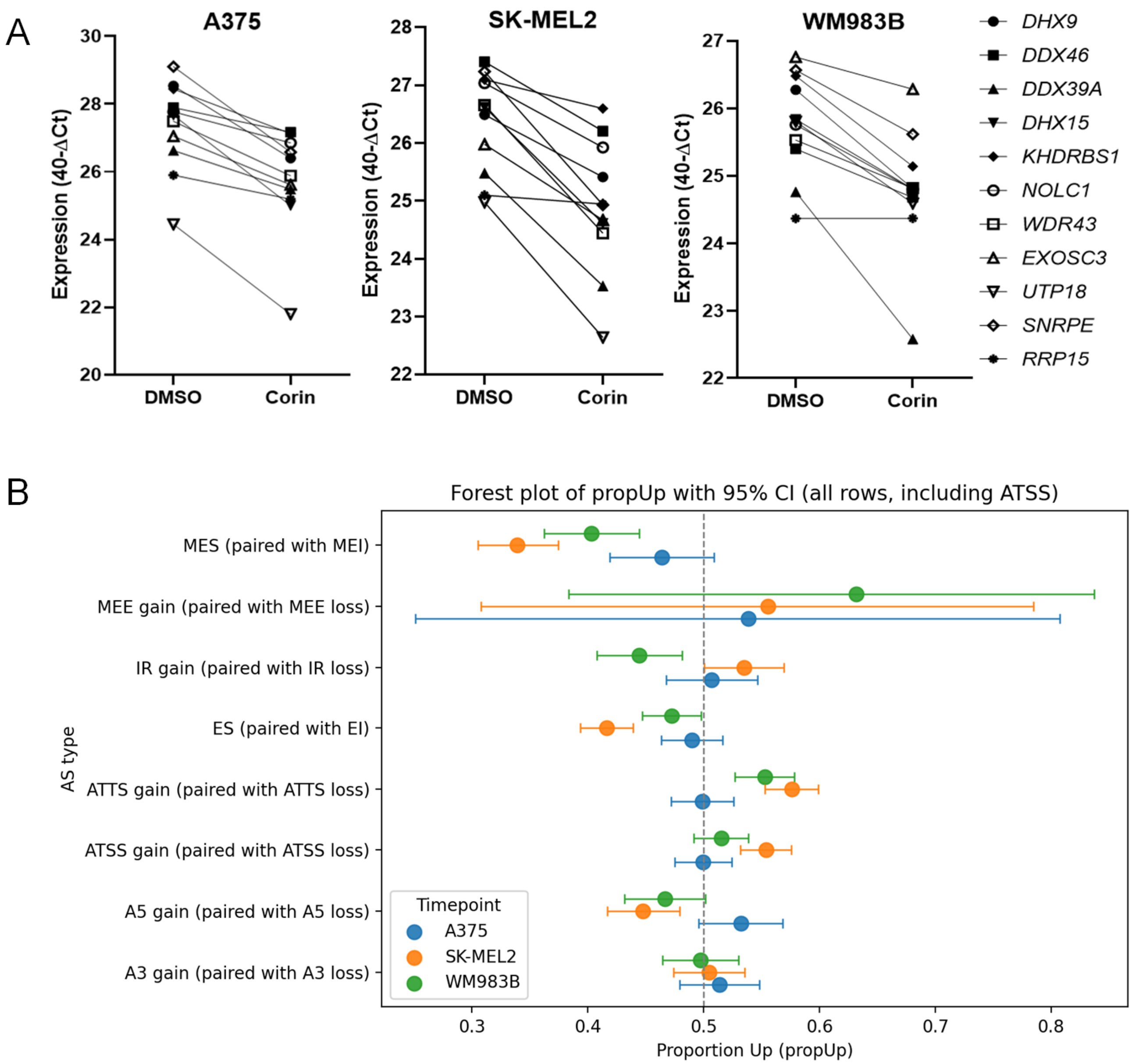

Figure S2. RNA processing genes are down regulated by CoREST complex inhibitor Corin. A) RT-PCR showing the downregulation of RNA processing genes following Corin treatment in A375, WM983B, and SK-MEL2 cell lines. **B)** Splicing analysis of significantly altered genes using IsoformSwitchAnalyzer on the RNAseq data comparing Corin-treated cells with DMSO controls. Alternative splicing events are classified as A3 (alternative 3' splice site), A5 (alternative 5' splice site), ATSS (alternative transcription start site), ATTS (alternative transcription termination site), ES (exon skipping), IR (intron retention), MEE (mutually exclusive exons), and MES (mutually exclusive splicing/exons).

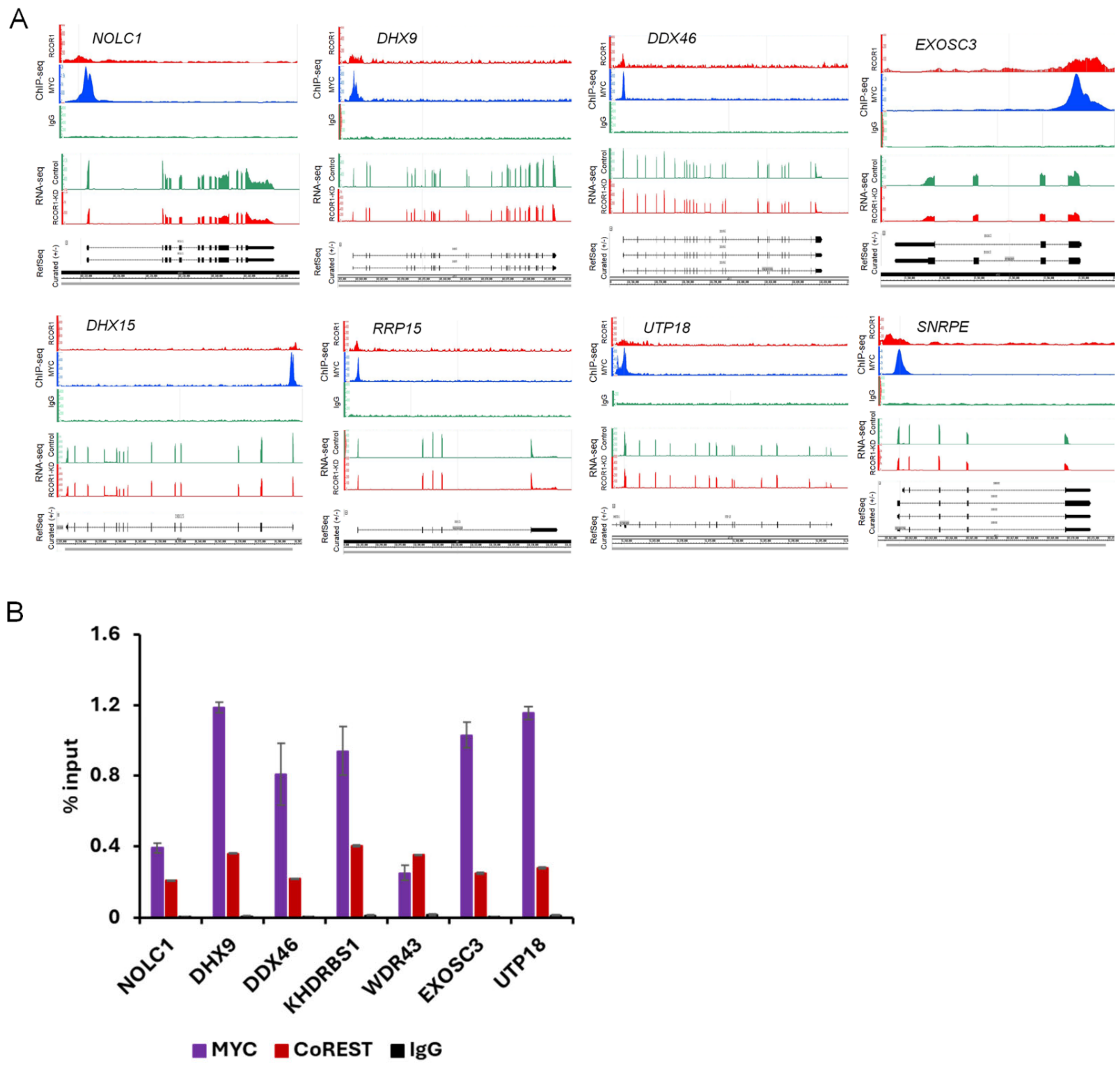

Figure S3. Direct transcriptional regulation of RNA processing genes by the CoREST complex and MYC in a melanoma cell line (A375). A) ChIP-seq peaks of the RCOR1 and MYC binding (top lanes) and RNA-seq (bottom lanes) at a group of selected gene promoters annotated with RNA processing (GOBP_RNA_PROCESSING, GO:0006396). **B)** The RCOR1 and MYC binding was confirmed by ChIP qPCR at a group of selected genes.

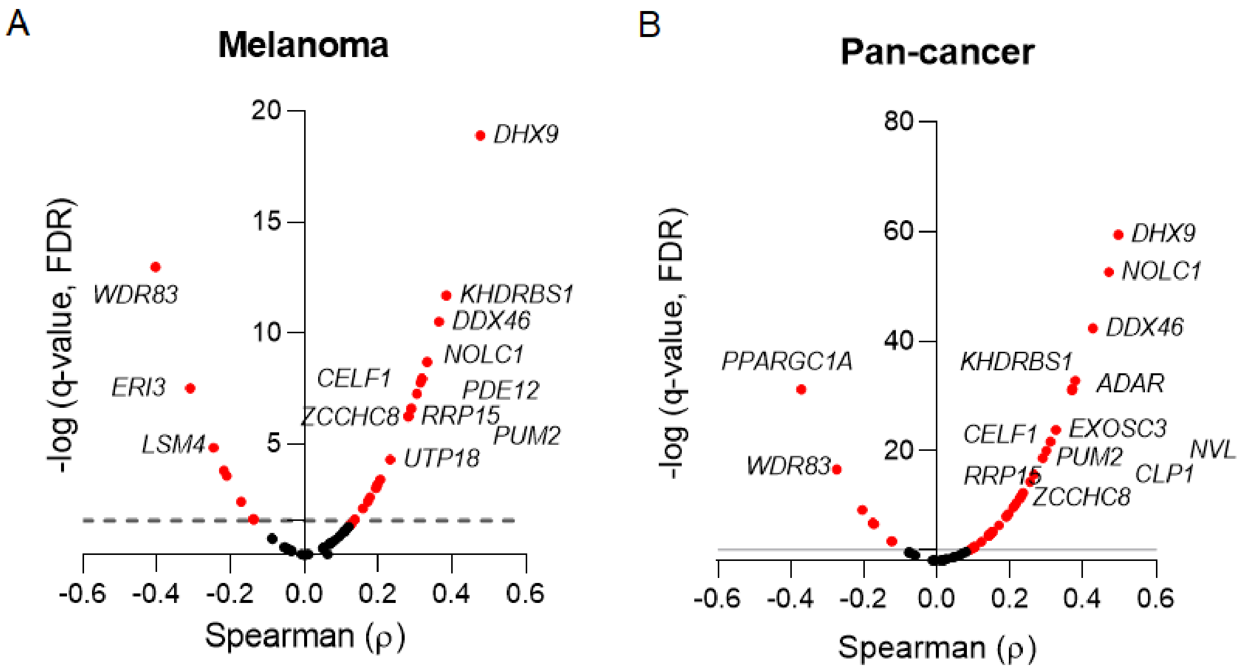

Figure S4. Expression correlation of RCOR1 with and RNA processing genes in patient samples. Spearman correlation coefficient (ρ) with statistical significances (q-value <0.001) between the expression of *RCOR1* and each of the 62 genes annotated as RNA processing and occupied by both RCOR1 and MYC in the promoter region. **A)** Patients with cutaneous melanoma (SKCM). **B)** All cancer types (Pan-cancer). TCGA datasets were used.

Table S1. Antibodies and primer sequences for RT-PCR, RTL-P, ChIP, and cloning for reporter vector.

RT-PCR	Gene name	Forward	Reverse
	MYC	GTCCTCGGATTCTCTGCTCTCC	CCTGCCTCTTTTCCACAGAAAC
	DHX9	GAGTTGACTCCTTTGGGACGAA	GAAAGCAGGTAGCAGCAGCAAT
	DDX46	GGTGAGCTGATGGAGAATGACC	CGCTGTTTTGTTTGATACCCTGT
	KHDRBS1	AGTACCTGAACCCTCTCGTGGA	GATGGCTCCCCTTACTGGTGTA
	NOLC1	ATGAGCCACCAAAGAACCAGAA	TGGCTGCTTTACCATTGGCTAT
	DDX39A	TGAGGACTCGGACACCTACCTG	GAGGATTTTGGCATCATTCTCG
	DHX15	TCCGGACATGTACAGATATCAAGC	GATGCGGTCCAACTGTCTCTTT
	WDR43	CTTTCACACCTTCATGGAAAGC	ACACCAACTTTGCCTTCTGTCC
	EXOSC3	CTCAGCAGAAGCGGTATGTTCC	GCTCACTCCCTCCAACATCAAC
	UTP18	TGACATGCTGGCTGGAAAGTTA	GCAATGCCATTTATGAGCAAGA
	SNRPE	AGATCGCGGATTCAGGTGTG	CATCAAAACCAATGATACAGCCTTC
	RRP15	TGAGAGGGATGGATGGAAGTACA	AAATCATCACGTAGGATCGTCCA
	STX1A	CAGTCCATCGAGCAAGAGGAAG	GGTTCCCACTCTCCAGCATGT
	FAM20C	GCTGAAGGTGCAGAATTCCTCT	AACAGCGCTTGCCCGTAAT
	GAPDH	ACCCACTCCTCCACCTTTG	CTCTTGTGCTCTTGCTGGG
	18srRNA	GTAACCCGTTGAACCCCATT	CCATCCAATCGGTAGTAGCG
**RTL-P**	U1 snRNA (*RNU1-1*)	F1: GAGATACCATGATCACGAAGGTGG<br>F2: GGATGTGCTGACCCCTGC	R: GGAAAGCGCGAACGCAGT
**ChIP**	*NOLC1*	GGCGGTTGGGAGATCTTGTAG	CTTTTCTCAGAGCAGCGGTTTC
	DHX9	TTGTCTCGGTAGAAGGCCAGAG	ATGGTAGCCGGAACGTCTCTC
	DDX46	GAAGAGGCGGGAGTTGAGGT	CCAAACACAACCATTCCCTCAG
	KHDRBS1	GAAACGAACGGAGCCCACT	CGGAGAACAAAGTCCCCAGTC
	WDR43	CCAGCCCACGGCTAATTTTT	CCTGAGGTCGGAGTTCGAGA
	EXOSC3	CTGTCCAAGGTGGCAGTGATT	AAGTGGAGGCTTTTCGATGGAC
	UTP18	AGCGGAGGAGACGAATGAAACT	CTTTCCAGTCCGGCCTCATT
	SNRPE	CCAGGGTCAGAAAGTGCAGAAG	CTGACCCGAACCTCCTAGTCCT
	RRP15	CAACTGTCAGGTGACGCTTCC	GAAGTGTCTGTGGCCTCGTCTT
	NOLC1-1	TATTCTGGGCGCTATGTGAGGT	GAGGCCATAGTGACACCCAAAG
	NOLC1-2	CAGCTTGGGAAATCAACACCTC	GAGGCCCTTTCTGATACCATCC
	NOLC1-3	AATAAGTTCGCCAAAGCGACAG	ACTTCCCCACCTGGAAACCTAA
**Cloning**	NheI_Nolc1_F1	GCTGCTAGCACTCATGACAAAGGCATTGATACTG	
	HindIII_Nolc1_R	TTTAAGCTTAGCTCCTGTCGCTTTGGCGAAC	
